# Utilization of palliative radiotherapy for bone metastases near end of life in a population-based cohort

**DOI:** 10.1186/s12904-015-0072-5

**Published:** 2016-01-10

**Authors:** Manpreet S. Tiwana, Mark Barnes, Andrew Kiraly, Robert A. Olson

**Affiliations:** BC Cancer Agency-Centre for the North, Prince George, Canada; University of Northern British Columbia, Prince George, Canada; University of British Columbia, Vancouver, Canada

**Keywords:** Bone metastases, Palliative, Radiation therapy, End of life

## Abstract

**Background:**

Palliative radiotherapy (PRT) can significantly improve quality of life for patients dying of cancer with bone metastases. However, an aggressive cancer treatment near end of life is an indicator of poor-quality care. But the optimal rate of overall palliative RT use near the end of life is still unknown. We sought to determine the patterns of palliative radiation therapy (RT) utilization in patients with bone metastases towards their end of life in a population-based, publicly funded health care system.

**Methods:**

All consecutive patients with bone metastases treated with RT between 2007 and 2011 were identified in a provincial Canadian cancer registry database. Patients were categorized as receiving RT in the last 2 weeks, 2–4 weeks, or >4 weeks before their death. Associations between RT fractionation utilization by these categories, and patient and provider characteristics were assessed through logistic regression.

**Results:**

Of the 16,898 courses 1734 (10.3) and 709 (4.2 %) were prescribed to patients in the last 2–4 weeks and <2 weeks of their life, respectively. Primary lung (8 %) and gastrointestinal (6.9 %) cancers received palliative RT more commonly in the last 2 weeks of life (OR 3.72 [2.86–4.84] & 3.33 [2.42–4.58] respectively, *p* <0.001). Among the 709 patients who received RT in the last 2 weeks of life, 350 (49), 167 (24), and 127 (18 %) were for spine, pelvis, and extremity metastases, respectively. RT was prescribed most frequently to spine (5 %) and extremity (4 %) metastases *p* <0.001 in the last two weeks of life, though only varied between 1 % (sternum) and 5 % (spine) by site of metastases. Single fraction RT was prescribed more commonly in the last 2 weeks of life (64.2 %), compared to individuals who received RT 2–4 weeks (54.5), and >4 weeks (47.9 %) before death (*p* <0.001).

**Conclusions:**

This population-based analysis found that only 4 % of patients with bone metastases received radiation therapy during the last 2 weeks of their life in our population-based, publicly funded program, though it was significantly higher in patients with lung cancer and those with metastases to the spine or extremity. Appropriately, use of multiple fractions palliative RT was less common in patients closer to death.

## Background

Approximately half of prescribed radiotherapy (RT) is delivered with palliative intent across North America [1, 2]. Palliative RT has numerous indications, and primarily includes the treatment of painful bone metastases [2]. Palliative RT for bone metastases provides successful pain relief, preservation of function, and maintenance of skeletal integrity with minimal risk of serious side effects [3, 4].

Palliative RT for bone metastases reduces pain in the majority of patients, though often takes several weeks [5]. The use of palliative RT use in the final weeks of life of therefore may have limited clinical use and may actually impair quality of life for patients and their families near the end of life [5].

The optimal rate of palliative RT use near the end of life is still unknown [5]. The use of systemic therapies at the end of life has been extensively studied, and same is warranted for optimal rate of palliative RT usage in such patients [6, 7]. An overly aggressive cancer treatment at the end of life may be an indicator of poor-quality care [6–8]. The total palliative RT dose, the dose per fraction and the technique of irradiation use may vary with the treatment aim in patients with bone metastases [9].

British Columbia (BC) provides 100 % of the radiation therapy in the province as a, publicly-funded service with no direct costs to patients. We previously published initial results demonstrating variation in RT prescribing practices for bone metastases in BC, where we demonstrated an association between fractionation and prognosis [10]. The primary objective of this current study is to explore the patterns of RT usage in patients with bone metastases towards their end of life. Understanding the patterns of palliative RT in patients with bone metastases will help us measure our quality and consistency of end of life care across the province.

## Methods

### Study design and cohort selection

This population-based retrospective study used administrative data to define a cohort of patients who received palliative RT for bone metastases during 2007 through 2011. This study was approved by the joint University of British Columbia and BC Cancer Agency (BCCA) Research Ethics Board.

### Data source and extraction

Patient data was extracted through the BCCA Cancer Agency Information System. The RT parameters were retrieved from BCCA’s RT database [11] and included site of RT, date of RT, dose, and fractionation. These databases were used to abstract patient, provider and treatment characteristics. Patient chart reviews, and review of RT plans where necessary, were performed to identify the various patient and physician related parameters associated with palliative RT in bone metastases. The provincial radiation therapy facilities up to 2011 were located in Abbotsford, Kelowna, Surrey, Vancouver & Victoria.

### Patient and treatment variables

The patients who received RT for bone metastases at the BC Cancer Agency from 2007 to 2011 were included in the analysis. The commonly occurring primary tumour sites were categorized as prostate, breast, lung, lymphoma, and gastro-intestinal (GI). The key sites of skeletal metastases were classified as spine, pelvis, extremity, rib, sternum, and ‘skull’, the latter of which included orbit and jaw. For descriptive analyses, RT fractionation was classified into single fraction (SF) or multiple fractions (MF).

### Statistical analysis

Time to death was calculated from last course of palliative RT. This time interval (in weeks) was categorized into three groups :< 2, and 2–4, and > 4 weeks. Association between these categories and the variables was analyzed through descriptive statistics, and chi-square test. Subsequently, univariable and multivariable linear regression analyses were performed to assess these associations. *P* values were two-sided, and values less than .05 were considered statistically significant. Analyses were conducted using the SPSS statistical software package, version 19.0 (Chicago, IL).

## Results

A total of 16,898 courses of palliative RT were delivered to 8301 patients from 2007 to 2011. Baseline patient and treatment related factors are summarized Table 1, and have also been presented in an earlier publication^10^. The median survival for the entire cohort was 18 weeks (95 % CI 17.49–18.51).

**Table 1:** Baseline patient, treatment, and provider characteristics

		Proportion	Proportion
		(Overall)	(Last 2 weeks of life)
		RT course, *n* = 16,898	RT course, *n* = 709
Age (years)	<51	8.1 %	8.3 %
	51–70	44.2 %	45.8 %
	>70	47.7 %	45.8 %
Male		50.2 %	47.5 %
Primary tumour	Prostate	19.0 %	10.2 %
	Breast	23.4 %	7.3 %
	Lung	22.4 %	42.0 %
	Hematological	11.2 %	8.0 %
	GI	7.8 %	12.8 %
	others	16.2 %	19.6 %
Skeletal metastasis	Spine	42.2 %	49.4 %
	Pelvis	28.6 %	23.6 %
	Extremity	17.1 %	17.9 %
	Ribs	8.1 %	6.6 %
	Sternum	1.7 %	0.4 %
	Skull	2.3 %	2.1 %
SFRT		49.2 %	64.2 %
BCCA centre	Abbotsford	5.6 %	5.9 %
	Kelowna	19.1 %	15.1 %
	Surrey	16.3 %	18.6 %
	Vancouver	35.8 %	33.7 %
	Victoria	23.2 %	26.7 %

*SFRT* single fraction radiation therapy, *BCCA* british columbia cancer agency

**Fig. 1:**
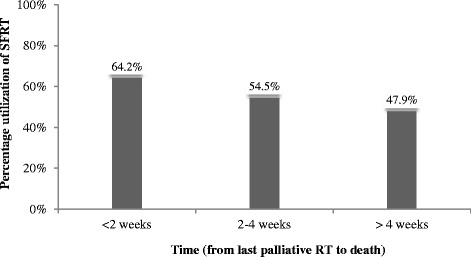
Percentage utilization of single fraction radiation therapy (SFRT), by time from last course of RT to death

Of the 16,898 courses 1734 (10.3) and 709 (4.2 %) were prescribed to patients in the last 2–4 weeks and <2 weeks of their life, respectively (Table 1). Table 2 highlights the univariate analysis on the utilization of palliative RT in the last 2 weeks of life. Single fraction RT was prescribed more frequently in patients with a shorter time from RT to end of life (Fig. 1).

**Table 2:** Clinical and provider characteristics associated with the use of palliative RT utilization in the last 2 weeks of life

Characteristic		Proportion who received RT in the last 2 weeks of life	*P* value
Age (years)	<51 (*n* =1364)	5.0 %	0.27
	51–70 (*n* =7477)	4.2 %	
	>70 (*n* =8057)	4.0 %	
Male (*n* =8491)		4.0 %	0.14
Primary tumour	Prostate (*n* =3218)	2.2 %	<0.001
	Breast (*n* =3959)	1.3 %	
	Lung (*n* =3777)	7.9 %	
	Hematological (*n* =1887)	3.0 %	
	Gastrointestinal (*n* =1319)	6.9 %	
	Other (*n* =2738)	5.1 %	
Skeletal metastasis	Spine (*n* =7134)	4.9 %	<0.001
	Pelvis (*n* =4826)	3.5 %	
	Extremity (*n* =2897)	4.4 %	
	Ribs (*n* =1362)	3.5 %	
	Skull (*n* =393)	3.8 %	
	Sternum (*n* =286)	1.0 %	
BCCA centre	Abbotsford (*n* =946)	4.4 %	0.01
	Kelowna (*n* =3221)	3.3 %	
	Surrey (*n* =2750)	4.8 %	
	Vancouver (*n* =6056)	3.9 %	
	Victoria (*n* =3925)	4.8 %	

*RT* radiation therapy, *BCCA* british columbia cancer agency

Multivariable logistic regression is presented in Table 3, demonstrating a significant association between use of RT in the last 2 weeks of life and site of primary, site of metastases, and BCCA Centre (Table 3). Lung cancer patients (*p* < 0.001), and those receiving RT to spinal metastases (*p* < 0.001), were more likely to receive RT in the last 2 weeks of life (Table 3).

**Table 3:** Multivariable logistic regression analysis on palliative RT utilization in the last 2 weeks of life

Characteristic		Odds ratio to receive RT in last 2 weeks of life (>1 favors RT)	95 % confidence interval	*P* value
Age of patient (continuous)		0.99	0.99–1.00	0.41
Patient gender	Female	Reference		
	Male	0.87	0.74–1.01	0.07
Primary tumour	Prostate	Reference		
	Breast	0.57	0.40–0.82	0.002
	Lung	3.72	2.86–4.84	<0.001
	Haematological	1.29	0.91–1.85	0.15
	Gastrointestinal	3.33	2.42–4.58	<0.001
	Others	2.35	1.76–3.14	<0.001
Skeletal metastasis	Spine	Reference		
	Pelvis	0.67	0.55–0.81	<0.001
	Extremity	0.87	0.71–1.08	0.21
	Ribs	0.66	0.48–0.90	0.01
	Sternum	0.22	0.07–0.69	0.01
	Skull	0.85	0.49–1.45	0.55
BCCA centre	Vancouver	Reference		
	Abbotsford	1.14	0.81–1.59	0.46
	Kelowna	0.86	0.68–1.09	0.20
	Surrey	1.29	1.09–1.62	0.03
	Victoria	1.34	1.09–1.62	0.005

*RT* radiation therapy, *BCCA* british columbia cancer agency

## Discussion

We demonstrated that 4 and 10 % of palliative RT courses were delivered to patients with bone metastases during the last 2 weeks, or last 2–4 weeks of their life, respectively in a large population based, publicly funded provincial RT program. Patients with lung and gastrointestinal cancers, or those receiving RT to their spine or extremity, were most likely to receive RT near the end of their life. Appropriately, longer multiple fraction (MF) RT courses were utilized less frequently for patients near their end of life.

Our finding of a 4 and 10 % utilization of palliative RT for bone metastases in last 2 or 2–4 weeks of life, though on the low end, are consistent with previous literature. The reported overall palliative RT utilization rates during the last 2 weeks of life are in the range of 2.2–14 % [2, 12–16]. Our relatively lower utilization rates of palliative RT for bone metastases in this study could be multifactorial, including accurate prognostication by treating physicians, a lack of financial incentive to offer RT in this publicly funded system, or patient’s choice to decline treatment [10, 17] Unfortunately, this cannot be assess in a retrospective study design.

Other authors state that the choice to offer palliative RT should be guided by life expectancy, though may also be influenced by a patient’s site of primary disease or area requiring palliation [18–20]. Indeed, we found a significant variation in the use of RT near the end of life, based on primary tumor site (most common for lung and GI cancer) and site requiring palliative RT (most common for spine and extremity) (Tables 2 and 3). We hypothesize the higher use of end of life RT in lung and GI cancers may be a factor of their worse prognosis, where treating physicians may be less accurately predicting their poor prognosis. Perhaps the use of prognostic indices could decrease the use of potential futile RT near the end of life, which unfortunately we cannot assess within this retrospective study [5, 21–23].

Furthermore, we hypothesize that the more frequent use of RT near the end of life for spine and extremity metastases is related to the severity of the symptoms they produce. As an example, physicians are likely more reluctant to withhold RT for a spinal cord compression or a fractured extremity, than they are for a fractured rib or painful sternum, irrespective of prognosis. We propose that physicians adopt more uniform use of prognostic tools before offering palliative RT, as it is unlikely that patients receiving RT in the last 2 weeks, even if they are suffering from a spinal cord compression or fractured extremity, given the required 2–4 weeks to see a clinical benefit. Finally, a more convenient and appropriate single fraction RT (SFRT) was prescribed in 64 % of the palliative RT courses to patients who died within 2 weeks of receiving RT. Multiple studies have shown and confirmed the benefit and efficacy of SFRT near end of life [18, 24, 25].

This study should be interpreted in the context of its strengths and limitations. Unfortunately, due to the retrospective nature of the study, information on patients’ cultural beliefs, their decision about treatment, hospital admission, or whether the bone metastases were complicated by fracture or neurological compromise was not available. Further, the efficacy of palliative RT in terms of pain control was also not analyzed due to the nature of study design. However, this population-based provincial study is relatively free from referral and selection bias, and choice of RT prescription is not influenced by physician remuneration or patient’s ability to pay in this public healthcare model with physicians on salary.

## Conclusions

This population-based analysis found that only 4 and 10 % of patients with bone metastases received radiotherapy during the last 2 weeks, or 2–4 weeks of their life, respectively. Radiotherapy near the end of life was used most frequently for lung and gastrointestinal cancers, potentially as a result of their inherently worse prognosis which physicians are not accurately predicting. End of life RT was also used frequently for patients receiving RT to the spine or extremity, which we hypothesize, is because of the potential severity of symptoms in these sites, such as spinal cord compression or fractured extremity. However, given the likely futility in offering RT during the last two weeks of life, our research supports the more widespread adoption of prognostic tool use prior to prescribing palliative RT. Appropriately, the use of multiple fractions palliative RT course was less frequently used in patients with a shorter lifespan.

## Abbreviations

RT: radiotherapy
BCCA: BC Cancer Agency
HSDA: health service delivery area
CAIS: cancer agency information system
OR: odds ratio
CI: confidence interval
SFRT: single fraction radiation therapy
GI: gastrointestinal
